# Coding-Complete Genome Sequences of Copenhagen and Copenhagen-Derived vP811 Strains of Vaccinia Virus Isolated from Cell Culture

**DOI:** 10.1128/mra.00090-23

**Published:** 2023-03-22

**Authors:** Mariann Landsberger, Joshua Quick, Jason Mercer

**Affiliations:** a MRC Laboratory for Molecular Cell Biology, University College London, London, United Kingdom; b Institute of Microbiology and Infection, School of Biosciences, University of Birmingham, Birmingham, United Kingdom; Queens College Department of Biology

## Abstract

The coding-complete genomes of laboratory vaccinia virus strain Copenhagen and the Copenhagen-derived deletion strain, vP811, were determined by short-read sequencing. Relative to the NCBI reference genome M35027, seven common coding differences were revealed, including an intact copy of the vaccinia virus immunomodulator A46R in both Cop and vP811.

## ANNOUNCEMENT

Vaccinia virus (VACV), a member of the *Orthopoxvirus* genus and *Poxviridae* family, was used to eradicate smallpox and vaccinate against monkeypox (1). The linear double-stranded DNA genome of VACV strain Copenhagen (Cop) is 191,636 bp and predicted to encode 263 genes (2) (M35027.1). The Cop deletion mutant vP811 was generated by deleting genes J2R, A26L, and A56R, replacing the region between the left inverted terminal repeat (ITR) and F4L with the Escherichia coli xanthine-guanine phosphoribosyltransferase gene, and swapping the region from B13L to the right ITR with Cop C7L (3). To date, neither the genome sequence of vP811 nor any updated Cop genome information has been deposited.

Laboratory Cop and vP811 stocks were obtained from the University of Alberta and stored in 1 mM Tris buffer, pH 9, at −80°C. Confluent monolayers of BSC-40 cells (ATCC CRL-2761) were infected at a multiplicity of infection of 0.02, and genomic viral DNA was obtained by phenol-chloroform extraction 3 days postinfection as described previously (4, 5).

Purified DNA was subjected to short-read sequencing (Genewiz, Germany). This included library preparation (NEBNext Ultra II DNA library preparation kit), sequencing with Illumina NovaSeq6000 using 2 × 150 paired-end configuration, and conversion of raw data into demultiplexed FASTQ files (bcl2fasq).

Default software parameters were used in the following procedures. Totals of 22,559,466 and 27,604,708 paired-end Cop and vP811 reads were randomly subsampled to 50,000 and 100,000 (Seqtk) (6). Subsampled Cop and vP811 reads had an average length of 144.6 and 140.4 nucleotides (nt) and mean quality scores of 35.4 and 35.35 (fastqcr) (7), respectively. They were used as input for *de novo* assembly in SPAdes v3.13.0 (8). Cop reads yielded a 167,603-nt contig with 25.2× and an 8,157-nt contig with 49.5× average sequencing coverage. The latter aligned to the coding region of the ITRs as determined by pairwise alignment with M35027 (Geneious Prime version 2022.1.1). The coding-complete Cop genome was assembled manually by adding ITR coding regions to the central genome. The assembly of vP811 reads resulted in a 134,690-nt contig with 20.7× average sequencing coverage. M35027 was used to annotate assemblies using the transfer annotation function and manually curated where necessary (Geneious).

The coding-complete genomic Cop and vP811 sequences consisted of 183,917 bp (33.5% GC) and 134,690 bp (33.4% GC), with 261 versus 184 predicted genes, respectively. Pairwise alignment with M35027 revealed 7 (Cop)/6 (vP811) intergenic or silent mutations, 14 (Cop)/11 (vP811) missense mutations, and frameshifts (Table 1). A 41-amino-acid (aa) deletion in A25L (vP811), not described in the initial study (3), was found to be a by-product of the A26L deletion process (9). Mapping of raw reads from two transcriptome sequencing (RNA-seq) studies to M35027 using minimap2 (10), SAMtools (11), IGV (12), and pairwise alignment of *de novo* assemblies with VACV strain Western Reserve (WR, AY243312.1) indicated that all differences in the Cop sample were present in the Cop virus used by Mehta et al. (13) and the Sementis Copenhagen vector and WR (14). The Toll-like receptor signaling antagonist A46R in both Cop and vP811 was identical to their functional WR homolog despite the original 1990 sequence encoding a truncated version (15, 16).

**TABLE 1:** Summary of identified changes in VACV Cop and vP811 genes compared to reference and presence in other related sequence submissions^*a*^

VACV Copenhagen strain M35027.1			Changes detected in:				
CDS	DNA sequence change	Amino acid change	Cop^*b*^	vP811^*b*^	WR (AY243312.1)	Copenhagen PRJNA468252 (13)	Sementis Copenhagen vector PRJNA610695 (14)
Left ITR-putative F4L	M35027.1:g.(?_37019)delinsAP009048.1:9.255977-256435 (inserted sequence consists of 300 noncoding nucleotides and *gpt* gene)	AAA47970.1_AAA48018.1delinsBAA77907.1:p.(Met1_Ter135)	NA	Yes (3)	NA	NA	NA
Putative C20L	M35027.1:g.6841_6842insT M35027.1:g.6844_6845insA	AAA47975.1:p.(Ile97TyrfsTer32)	Yes	NA	Yes (AAO89286.1)	Yes	Yes
Putative C2L	M35027.1:g.23443C>T	AAA47999.1:p.(Arg238=)	Yes	NA	Yes (AAO89305.1)	Yes	Yes
Putative C1L	M35027.1:g.24256G>C	AAA48000.1:p.(Arg215=)	Yes	NA	Yes (AAO89306.1)	Yes	Yes
Putative N2L	M35027.1:g.25525G>C	AAA48002.1:p.(Pro121Arg)	Yes	NA	Yes (AAO89308.1)	Yes	Yes
Putative F3L	M35027.1:g.35080_35081delinsGC	AAA48016.1:p.(Thr313Ser)	Yes	NA	Yes (AAO89321.1)	Yes	Yes
Putative F ORF A	M35027.1:g.35080_35081delinsGC	AAA48017.1:p.(Pro48=); AAA48017.1:p.(Val49Leu)	Yes	NA	NA^*c*^	Yes	Yes
Putative F13L	M35027.1:g.44312A>G	AAA48031.1:p.(Asp256=)	Yes	Yes	Yes (AAO89331.1)	Yes	Yes
Putative F16L	M35027.1:g.46742_46743delinsGC	AAA48035.1:p.(Arg10Ala)	Yes	Yes	Yes (AAO89334.1)	Yes	Yes
Putative I8R	M35027.1:g.70239G>T	AAA48064.1:p.(Val56Leu)	No	Yes	No (AAO89356.1)	No	No
Putative G7L	M35027.1:g.77258A>T	AAA48071.1:p.(Val348Glu)	Yes	Yes	Yes (AAO89364.1)	Yes	Yes
Putative L3L	M35027.1:g.81836G>A	AAA48078.1:p.(Leu138Phe)	Yes	Yes	Yes (AAO89369.1)	Yes	Yes
Putative J2R	M35027.1:g.83858_84385delins TAATTAACTAGCTACCCGGGTAAGTAATACGTCAAGGAGAAAACGAAACGATCTGTAGTTAGCGGCCGCCTAATTAAC	AAA48082.1:p.(0)	NA	Yes (3)	NA	NA	NA
Putative J3R	M35027.1:g.85139T>C	AAA48083.1:p.(Val229Ala)	Yes	Yes	Yes (AAO89374.1)	Yes	Yes
Putative D6R	M35027.1:g.104656C>T	AAA48105.1:p.(Thr280Met)	Yes	Yes	Yes (AAO89390.1)	No reads	Yes
Putative D11L	M35027.1:g.109374G>T	AAA48110.1:p.(Leu357Ile)	No	Yes	No (AAO89395.1)	No	No
Putative A24R	M35027.1:g.134579G>T	AAA48148.1:p.(Gly89Cys)	No	Yes	No (AAO89423.1)	No	No
Putative A25L	M35027.1:g.137889_138947del	AAA48150.1:p.(0)	NA	Yes (3, 9)	NA	NA	NA
Putative A26L	M35027.1:g.137889_138947del	AAA48151.1:p.(0)	NA	Yes (3)	NA	NA	NA
Putative A41R	M35027.1:g.148904A>T	AAA48172.1:p.(Thr84=)	Yes	Yes	Yes (AAO89445.1)	Yes	Yes
Putative A46R	M35027.1:g.152699_152700insC	AAA48177.1:p.(Asn186GlufsTer55)	Yes	Yes	Yes (AAO89451.1)	Yes	Yes
Putative A ORF Q	M35027.1:g.152699_152700insC	AAA48178.1:p.(Ala3_Asn4delinsGlyTer)	Yes	Yes	NA^*c*^	Yes	Yes
Putative A54L	M35027.1:g.158626del	AAA48187.1:p.(Ile40Ter)	No	Yes	No^*c*^	No	No
Putative A56R	M35027.1:g.161185_162053delins GATCTCCCGGGCTGCAG	AAA48191.1:p.(0)	NA	Yes (3)	NA	NA	NA
Putative A ORF U	M35027.1:g.161185_162053delins GATCTCCCGGGCTGCAG	AAA48192.1:p.(0)	NA	Yes (3)	NA	NA	NA
Putative B26R	M35027.1:g.184893_184894insT; M35027.1:g.184896_184897insA	AAA48226.1:p.(Ile97TyrfsTer32)	Yes	NA	Yes (AAO89491.1)	Yes	Yes
Putative B13R-right ITR	M35027.1:g.(172549_?)delins19258_18805 (inserted sequence consists of 308 noncoding nucleotides and C7L gene)	AAA48210.1_AAA48229.1delinsAAA47993.1:p.(Met1_Ter151)	NA	Yes (3)	NA	NA	NA

a CDS, coding sequence; ORF, open reading frame; NA, not available.

b This study.

c Not annotated in WR (16).

### Data availability.

Sequences have been deposited in GenBank under accession numbers OP868847 and OP868848. The raw reads were deposited in the NCBI Sequence Read Archive (SRA) under the accession numbers PRJNA902654, SRR22318734, and SRR22318733.
